# Leptospiral shedding and seropositivity in shelter dogs in the Cumberland Gap Region of Southeastern Appalachia

**DOI:** 10.1371/journal.pone.0228038

**Published:** 2020-01-30

**Authors:** Dawn Spangler, Daniel Kish, Brittney Beigel, Joey Morgan, Karen Gruszynski, Hemant Naikare, Vinayak K. Nahar, Michele D. Coarsey, Ashutosh Verma

**Affiliations:** 1 College of Veterinary Medicine, Lincoln Memorial University, Harrogate, TN, United States of America; 2 Center for Infectious, Zoonotic and Vector-borne Diseases, Harrogate, TN, United States of America; 3 Center for Human and Animal Health in Appalachia, Lincoln Memorial University, Harrogate, TN, United States of America; 4 Tifton Veterinary Diagnostic and Investigational Lab, College of Veterinary Medicine, University of Georgia, Tifton, GA, United States of America; 5 Department of Dermatology, School of Medicine, The University of Mississippi Medical Center, Jackson, MS, United States of America; 6 Department of Preventive Medicine, School of Medicine/John D. Bower School of Population Health, The University of Mississippi Medical Center, Jackson, MS, United States of America; Bharathidasan University, INDIA

## Abstract

**Background:**

Leptospirosis, caused by pathogenic *Leptospira* spp., is a zoonotic infection that affects humans, dogs and many other mammalian species. Virtually any mammalian species can act as asymptomatic reservoir, characterized by chronic renal carriage and shedding of a host-adapted leptospiral serovar. Environmental contamination by chronic shedders results in acquisition of infection by humans and susceptible animals.

**Methods:**

In this study, we investigated if clinically normal shelter dogs and cats harbor leptospires in their kidneys by screening urine samples for the presence of leptospiral DNA by a TaqMan based-quantitative PCR (qPCR) that targets pathogen-associated *lipl32* gene. To identify the infecting leptospiral species, a fragment of leptospiral *rpoB* gene was PCR amplified and sequenced. Additionally, we measured *Leptospira*-specific serum antibodies using the microscopic agglutination test (MAT), a gold standard in leptospiral serology.

**Results:**

A total of 269 shelter animals (219 dogs and 50 cats) from seven shelters located in the tri-state area of western Virginia, eastern Tennessee, and southeastern Kentucky were included in this study. All cats tested negative by both qPCR and MAT. Of the 219 dogs tested in the study, 26/198 (13.1%, 95% CI: 8.4–17.8%) were positive for leptospiral DNA in urine by qPCR and 38/211 (18.0%, 95% CI: 12.8–23.2%) were seropositive by MAT. Twelve dogs were positive for both qPCR and MAT. Fourteen dogs were positive by qPCR but not by MAT. Additionally, leptospiral *rpoB* gene sequencing from a sub-set of qPCR-positive urine samples (n = 21) revealed *L*. *interrogans* to be the leptospiral species shed by dogs.

**Conclusions:**

These findings have significant implications regarding animal and public health in the Cumberland Gap Region and possibly outside where these animals may be adopted.

## Introduction

Leptospirosis, caused by pathogenic *Leptospira* spp., is a waterborne zoonotic infection that affects dogs and many other mammalian species [1,2,3]. Leptospires live in the proximal renal tubules of reservoir animals and are shed in the urine. The infection is contracted either through direct contact to urine of an infected animal or indirectly by exposure to *Leptospira*-contaminated water [4,5]. Leptospiral infection in dogs can result in a serious clinical outcome, such as acute hepatorenal failure, or it can also lead to asymptomatic chronic carrier state [6]. Chronic carriers may act as a source of infection and, for this reason, are of public health concern.

The prevalence of canine leptospirosis is increasing throughout the US, but some regions are considered hotspots of leptospirosis due to disproportionately large clusters of cases [7,8,9,10,11,12]. The Cumberland Gap Region (CGR), located close to the intersection of the state boundaries of Kentucky, Tennessee, and Virginia, is primarily a rural area that has all climatic, topographical, and socioeconomic factors that have been described as risk predictors for the occurrence of leptospirosis [13]. In a previous study, canine leptospirosis testing data collected over a period of 14 years in the United States was analyzed to develop predictive models for identifying regions of increased risk for leptospirosis. In that study, several counties in Appalachia had predictive probabilities for dogs testing seropositive. But the CGR was underrepresented in the testing data, likely due to the poor socio-economic status of the communities and a lack of veterinary care for pets in this region [13].

Since no information was available regarding prevalence of canine or feline leptospirosis in the region, we tested dogs and cats from seven shelters across three states in the CGR for the presence of leptospiruria and leptospiral antibodies. Leptospiral shedding was tested by screening urine samples for the presence of leptospiral DNA using a highly sensitive and specific TaqMan-based qPCR. In addition, dogs and cats were screened for the presence of leptospiral antibodies using microscopic agglutination test, a serodiagnostic gold standard.

Shelter dogs and cats are sentinels for many zoonotic diseases, likely due to unsanitary living conditions, high population density, stress, and exposure to rodents and other disease vectors. Here, we present our findings of leptospiral shedding and seropositivity among shelter animal populations in the CGR.

## Materials and methods

### Sample collection and DNA extraction

**Fig 1:**
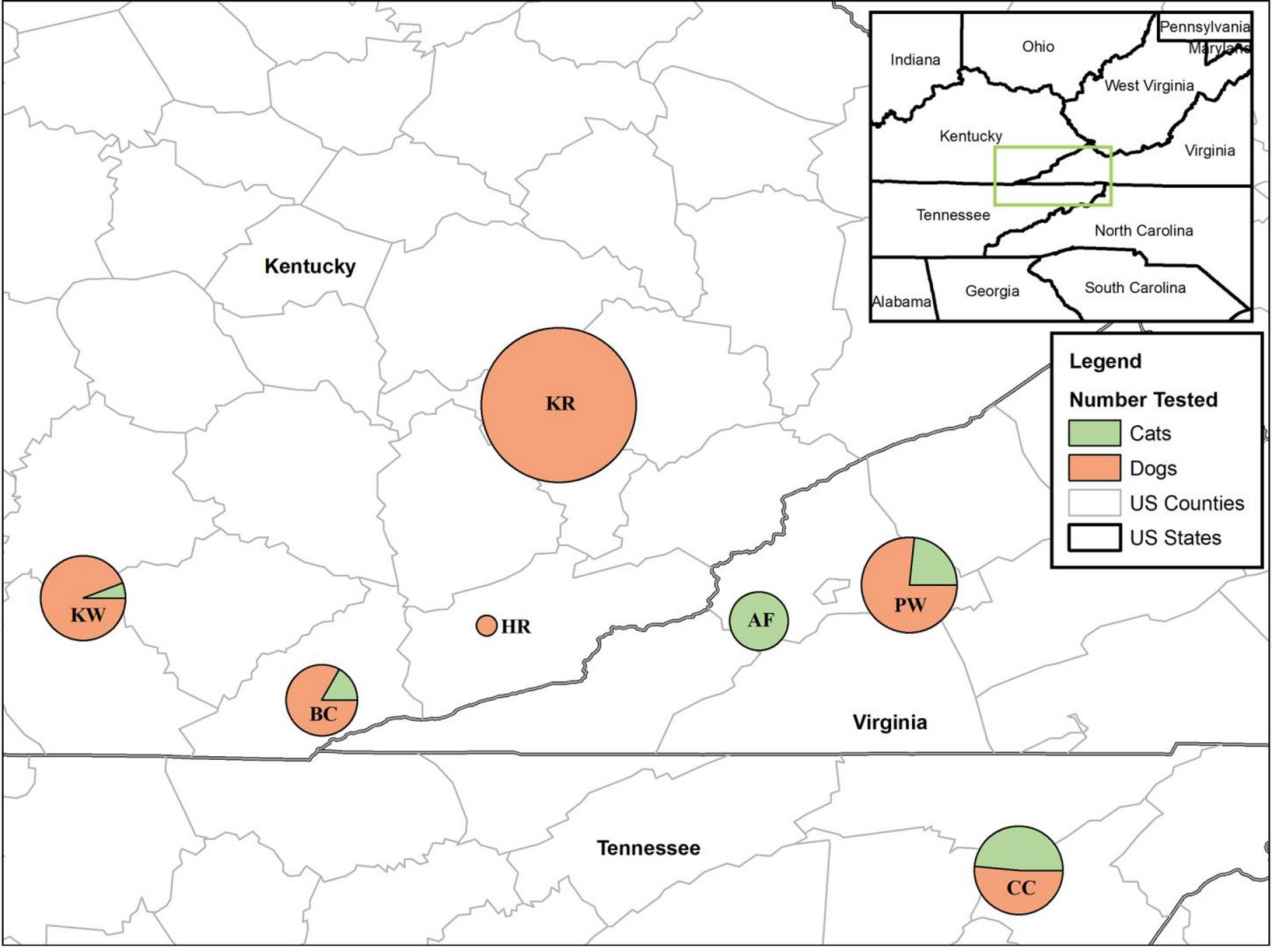
Map depicting the proportion of cats and dogs from seven shelters in Kentucky, Tennessee, and Virginia that were tested in this study. Samples were collected from 113 animals (all dogs) from Shelter KR, 34 animals (32 dogs and 2 cats) from Shelter KW, 24 animals (20 dogs and 4 cats) from Shelter BC, 2 animals (both dogs) from Shelter HR, 43 animals (33 dogs and 10 cats) from Shelter PW, 16 animals (all cats) from Shelter AF, and 37 animals (19 dogs and 18 cats) from Shelter CC. Map created with ArcMap 10.6 (Esri, Redlands, CA).

Dogs and cats from seven shelters located in three states (Tennessee, Kentucky and Virginia) were sampled in this study from April 2017 to Mar 2018 (**Fig 1**). Blood and urine samples were collected shortly after their arrival at the Lincoln Memorial University—College of Veterinary Medicine’s Small Animal Medical Center under the Shelter Outreach for the Appalachian Region (SOAR) program. The SOAR program provides spay/neuter services and veterinary care to unowned animals. These animals were clinically healthy and the number of animals included in the study from each shelter depended solely on the number of animals that were brought to the LMU-CVM Small Animal Medical Center for spay/neuter and basic veterinary care.

Blood samples (1.5mL) were drawn by venipuncture and collected in IDEXX Vacuette SST-Serum Separator Tube. Tubes were then centrifuged (2,000 g for 10 min) and serum pipetted off, aliquoted and stored at -20°C. Urine was collected by free-catch method. Demographic data including sex, age, breed, and shelter were recorded in Excel (Microsoft, Redmond, WA). Additional information regarding blood urea nitrogen (BUN; Azostick®) results and month of sample collection were also recorded. Up to 1.5 mL of urine sample was centrifuged at 17000 g for 5 min, supernatant discarded and DNA from pellet extracted using the PureLink Genomic DNA Mini Kit (Invitrogen). Extracted DNA was stored at -20°C. *Leptospira interrogans* serovar Pomona was grown in Polysorbate-80 bovine serum albumin medium (NVSL) at 30°C, and genomic DNA was isolated and quantified as previously described [14] for use as a control in qPCR.

#### Quantitative Polymerase Chain Reaction (qPCR)

We used a TaqMan based quantitative PCR (qPCR) to target a 242 bp region of leptospiral *lipl32* gene, as previously described [15]. The assay was performed in a MicroAmp Fast Optical 96-well reaction plate (Applied Biosystems, Foster City, CA, USA). Standard curve was created using DNA equivalent to 10^7^, 10^6^, 10^5^, 10^4^, 10^3^, 10^2^, 10, 1 leptospiral genome units. Each column, except positive control columns, had a no-template control. Each reaction was performed in a 25 μL final volume, using 5 μL of extracted DNA, 500 nM of LipL32-45F (forward primer; 5ꞌ-AAGCATTACCGCTTGTGGTG-3ꞌ), 500 nM of LipL32-286R (reverse primer; 5ꞌ-GAACTCCCATTTCAGCGATT-3ꞌ) and 100 nM of LipL32-189P (probe; FAM-5ꞌ-AAAGCCAGGACAAGCGCCG-3ꞌ-BHQ1) [15]. The assay was performed on a QuantStudio 3 using Platinum Quantitative PCR SuperMix-UDG (Invitrogen, Carlsbad, CA, USA) and thermal conditions of a holding stage of 95°C for 20 s, and 40 cycles of 95°C for 3 s and 60°C for 30 s. Each sample was tested in duplicate and repeated at least twice.

#### Microscopic agglutination test

Microscopic agglutination test was performed following OIE protocol (https://www.oie.int/fileadmin/Home/eng/Health_standards/tahm/3.01.12_LEPTO.pdf). Two-fold serum dilutions from 1:100 to 1:6400 were tested against serovars Pomona, Hardjo, Grippotyphosa, Icterohaemorrhagiae, Canicola, Bratislava and Autumnalis. The titer was defined as the reciprocal of the highest dilution of a serum sample that agglutinated more than half of leptospires. Titers of more than or equal to 1:100 were considered positive for the presence of leptospiral antibodies.

### Leptospiral *rpoB* gene sequencing

PCR amplification and sequencing of a fragment of leptospiral *rpoB* gene was performed for all positive urine samples as described previously [16]. Briefly, DNA from all qPCR-positive samples were subjected to PCR amplification of a 600bp fragment of *rpoB* gene using a Phusion High Fidelity polymerase (Thermofisher, Waltham, MA), primers Lept 1900f (5’-CCTCATGGGTTCCAACATGCA-3’) and Lept 2500r (5’-CGCATCCTCRAAGTTGTAWCCTT-3’), and thermal conditions as described previously [16]. PCR amplicons were sequenced at a commercial sequencing facility (Davis sequencing, Davis, CA), and compared to available sequences by BLAST search using the National Center for Biotechnology Information server (http://www.ncbi.nlm.nih.gov/BLAST/). Phylogenetic analyses were performed by the Neighbor-Joining method [17] using Geneious 9.0.5 software and phylogenetic distances measured by Tamura-Nei model.

#### Statistical analysis

Statistical analysis was performed on data using IBM SPSS Statistics 24 (IBM, New York) and Epi Info 7.2.2.6 (CDC, Atlanta, GA). Briefly, Chi-square tests or Fisher’s exact tests were performed for the variables: sex, breed, season, and shelter with test results from the urine qPCR and serum MAT. Odds ratios with 95% confidence levels were also calculated for each variable. In addition, Chi-square tests were used to determine individual shelter differences. A kappa test was performed to determine agreement between the qPCR and MAT tests.

#### Ethics statement

All animal experiments were carried out in strict accordance with the recommendations in the Animal Welfare Act of 1966, its amendments and associated Regulations (https://www.nal.usda.gov/awic/animal-welfare-act). All protocols were reviewed and approved by the Institutional Animal Care and Use Committee at the Lincoln Memorial University (protocol number: 1703-RES-04).

## Results

A total of 269 animals (219 dogs and 50 cats) from seven shelters located in the Cumberland Gap region of KY, TN and VA were included in this study. Blood and urine samples (blood and urine from 229 animals, only blood from 25 animals and only urine from 15 animals) were collected from shelter animals shortly after their arrival at the LMU-CVM Small Animal Medical Center. Blood and/or urine samples were collected from 113 animals (all dogs) from Shelter KR, 34 animals (32 dogs and 2 cats) from Shelter KW, 37 animals (19 dogs and 18 cats) from Shelter CC, 43 animals (33 dogs and 10 cats) from Shelter PW, 2 animals (both dogs) from Shelter HR, 16 animals (all cats) from Shelter AF, and 24 animals (20 dogs and 4 cats) from Shelter BC (**Fig 1**).

**Fig 2:**
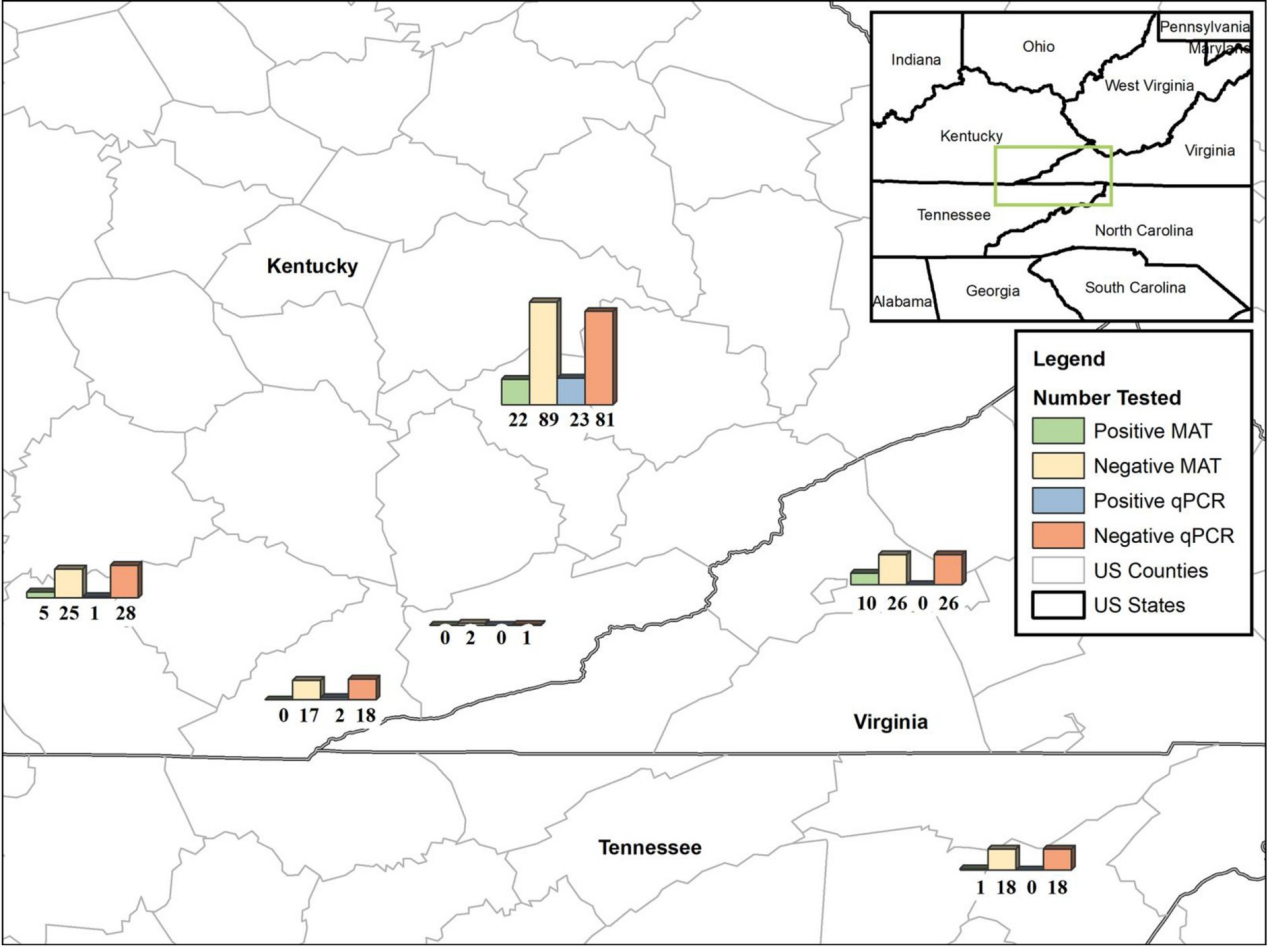
Map depicting MAT and qPCR test results for dogs from six shelters in Kentucky, Tennessee, and Virginia that were tested in this study. A seventh shelter, which only housed cats, is not included in the figure. Map created with ArcMap 10.6 (Esri, Redlands, CA).

Out of 269 dogs and cats included in this study, we were able to collect urine samples from 244 animals (198 dogs and 46 cats). A TaqMan based qPCR that targets *lipl32* gene of pathogenic *Leptospira* was used to screen DNA extracted from urine samples. Twenty six of 198 tested dogs (13.1%, 95% CI: 8.4–17.8%) were positive by qPCR (**Table 1**). Positive urine samples contained between 0.72 x 10^3^–0.24 x 10^4^ leptospiral genomic units/1.5ml of urine. Of 26 qPCR-positive dogs, 23 came from Shelter KR, 2 were from Shelter BC and 1 dog came from Shelter KW (**Fig 2**). All urine samples from cats tested negative for the presence of leptospiral DNA by qPCR.

**Table 1:** Prevalence of *Leptospira* spp. in shelter dogs and cats in the Cumberland Gap Region of Southeastern Appalachia.

Sample Type	Dog Sera tested	Positive dog sera (percent; 95% CI)	Cat sera tested	Positive cat sera (percent; 95% CI)	Test
**Urine**	198	26 (13.1%; 8.4–17.8%)	46	0	qPCR
**Blood**	211	38 (18.0%; 12.8–23.2%)	43	0	MAT

Using DNA extracted from qPCR positive urine samples, a 600 bp fragment of leptospiral *rpoB* gene was PCR amplified and sequenced. Twenty-one urine samples yielded good quality *rpoB* gene sequences. *rpoB* nucleotide sequences deposited in the GenBank have accession numbers MN731621-MN731641 (**S1 Table**). Analysis of these sequences revealed >99% homology with *rpoB* gene fragments of *L*. *interrogans* homologous gene fragments. By phylogenetic analysis, the *rpoB* genes of leptospiral strains MarleyKR, MarbleKR, DozerKR, LewisKR, BarneyKR, DarlaKR, HossKR, MirandaKR, BrandiKR, MaryAnnKR, ArielKR, JupiterKR, MandyKR, CindiKR, DaisyKR, GraceKR, HankKR, MeekoKR, CaliKR, HoldenKR and LandonKR (**S1 Table**) clustered very closely with the cognate gene of *L*. *interrogans* strains (**S1 Fig**). *L*. *interrogans* serovar Lai strain 56601 appeared to be the nearest neighbor of all leptospiral strains identified in this study, except Landon KR, which was the nearest neighbor to *L*. *interrogans* serovar Bataviae (**S1 Fig**).

Blood samples were drawn from 254 animals (211 dogs and 43 cats). Sera were tested for leptospiral antibodies using microscopic agglutination test (MAT). Of 211 dog sera tested, 38 contained *Leptospira*-specific antibodies (18.0%, 95% CI: 12.8–23.2%) (**Table 1**). Fifty per cent of the positive sera (19/38) reacted with only one of the seven tested serovars, with the majority (n = 11) being reactive to serovar Icterohaemorrhagiae, followed by Autumnalis (n = 4), Bratislava (n = 4), and Hardjo (n = 1) (**Table 2**). The remaining positive sera (n = 19) reacted to 2 (n = 7), 3 (n = 6), 4 (n = 4) or 5 (n = 2) serovars. Shelter KR had the highest number of MAT-positive dogs (n = 22), followed by Shelter PW (n = 10), Shelter KW (n = 5) and Shelter CC (n = 1) (**Fig 1**). None of the cats were positive by MAT and were excluded from further analyses.

**Table 2:** Seroreactivity of microscopic agglutination test (MAT)-positive dogs.

Sample ID	Pomona	Hardjo	Grippotyphosa	Ictero.^a^	Canicola	Bratislava	Autumnalis
**18**							100
**23**							100
**27 ^P^**				1600	100	400	200
**28**				100			
**36**				200		400	
**39 ^P^**							200
**42 ^P^**				400		200	200
**44 ^P^**				400		400	200
**45 ^P^**				1600		400	200
**55**				100			
**57 ^P^**				800		400	400
**58**						100	
**59 ^P^**				200			
**60 ^P^**				800	100		
**66 ^P^**				800	100		
**67 ^P^**				800			
**70 ^P^**				100			
**76**						400	
**89**				100			
**102**				100			
**104**				200			
**109**		100					
**115**	100		100	400		400	400
**116**	100			400		200	400
**130**	200			400		200	400
**139**				100			100
**140 ^P^**						100	
**149**				100			
**166**				400	100	100	100
**172**				100			200
**173**				400			
**176**				100			100
**180**				100		100	
**181**				100	100	100	
**182**			200			100	200
**188**				200			
**189**							200
**194**	100		400	200		800	400

a, Icterohaemorrhagiae

P, positive for both MAT and urine qPCR

Twelve dogs were positive by both qPCR and MAT. Ten of these 12 dogs exhibited highest antibody titers for serovar Icterohaemorrhagiae, eight of which had a titer of ≥ 400. Fourteen dogs were positive by qPCR but negative by MAT.

**Fig 3:**
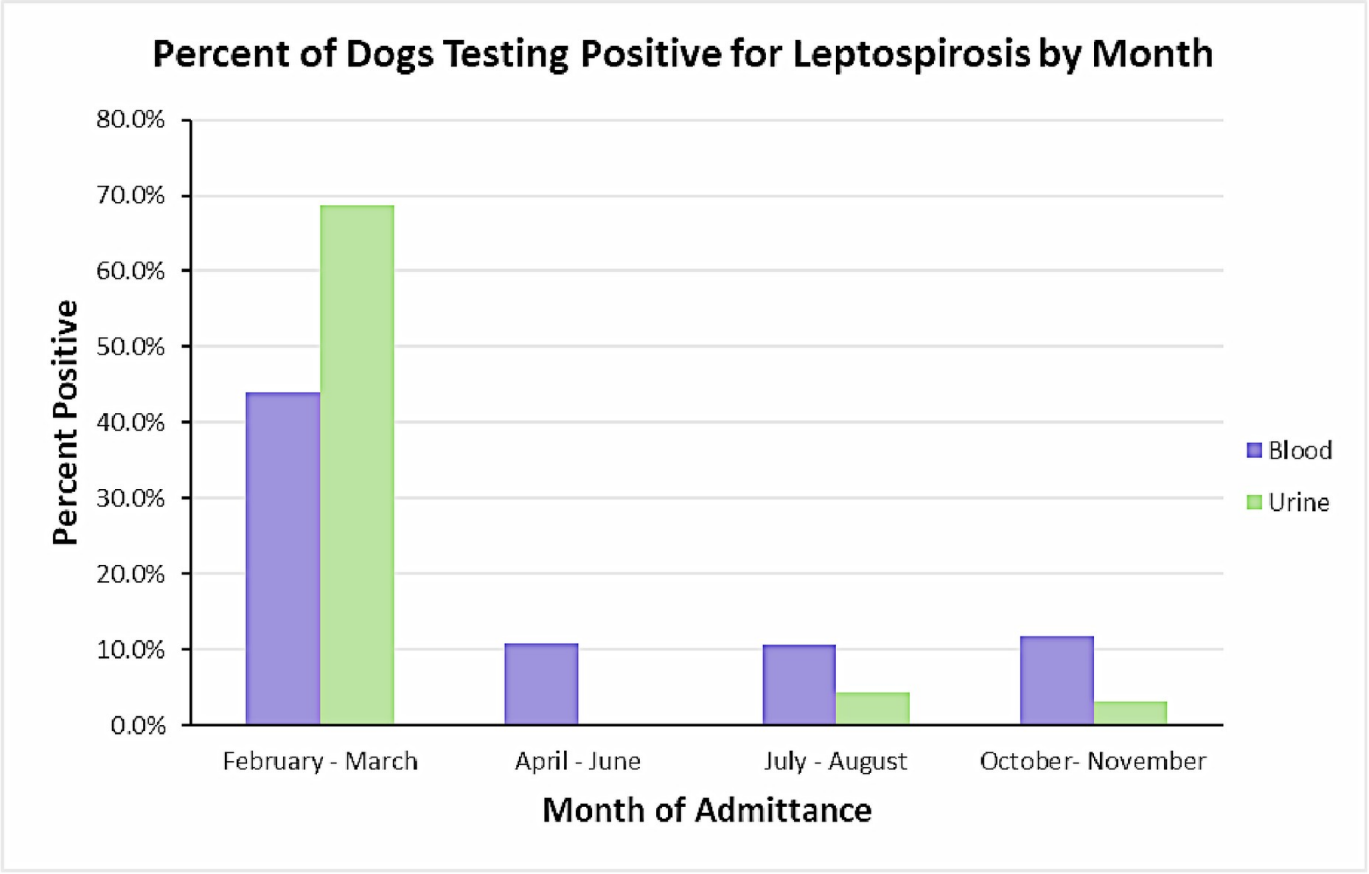
The percent of dog samples that tested positive for leptospiruria (urine) or leptospiral antibodies (blood) by month of admittance to the Lincoln Memorial University’s SOAR program. Of note, animals were not admitted in January, September, and December.

No analysis was performed on BUN results as all dogs in the study were within the normal range. Age was also dropped from analysis due to some validity concerns. The 95% CI for the variables sex and breed crossed the 1.0 threshold indicating there were no observed differences between the groups (**Table 3**). However, dogs admitted between February and June were 9.23 (95% CI: 3.04–28.02) times more likely to be qPCR positive and 2.66 (95% CI: 1.22–5.55) times more likely to be MAT positive than dogs admitted between July and November (**Table 3**). Further analysis of the variable season demonstrated statistically significant differences in the proportion of dogs admitted in Feb-March, April-June, July-August, and Oct-November (p<0.01) testing positive. In general, dogs were more likely to be positive earlier in the year than from summer through winter (p<0.01; **Fig 3**).

**Table 3:** Variable analysis of data collected from dogs admitted to LMU SOAR program. Results stratified by diagnostic test performed.

Test	Variable		Positive	Total	Percent positive	Odds ratio	95% CI
**qPCR**	Sex	Female	12	90	13.3%	1.03	0.45–2.36
		Male	14	108	13.0%	-	
	Breed*	Mixed	23	159	14.5%		0.49–9.64
		Other	3	34	8.8%	-	
	Season	February-June	22	84	26.2%	9.23	3.04–28.02
		July-November	4	104	3.8%	-	
**MAT**	Sex	Female	16	99	16.2%	0.78	0.38–1.59
		Male	22	111	19.8%	-	
	Breed	Mixed Other	33 4	172 28	19.2% 14.3%	1.66 -	0.55–5.06
	Season	February-June	24	97	24.7%	2.6	1.22–5.55
		July-November	12	106	11.3%	-	

* Odds Ratio not calculated due to < 5 as an expected value in a cell.

A Chi-square test was performed to identify differences among shelters in relation to test results. A statistically significant difference was noted among the shelters in relation to the qPCR results (p<0.01). Shelter KR had 23 of 26 qPCR-positive dogs. For MAT results, no differences were found among shelters (p-value = 0.07). Kappa test statistic was performed to find if there was an agreement between qPCR and MAT results. Our data from qPCR and MAT produced an agreement of 0.33 (p<0.01), indicating a fair agreement.

## Discussion

Pet overpopulation is an emerging issue in the Appalachian region relative to the rest of the United States, and this problem is likely attributed to various cultural and socio-economic factors unique to the region [18,19]. Stray animals have a higher exposure to rodents and contaminated standing water, which may be the only survival strategy available to many stray animals prior to adoption or arrival at an animal shelter. Once at a shelter, crowding, unsanitary housing conditions, and a lack of proper veterinary care further put these animals at a risk of getting infected, becoming carriers and transmitting diseases to both animals and humans.

In the present study, 13.1% of 219 tested apparently healthy dogs had leptospiral DNA in their urine indicating shedder status of these animals. Previous studies have shown that clinically normal dogs can chronically shed leptospires in urine, contaminating the surroundings and potentially exposing other animals and people in that environment to infection [20,21,22,23]. Identification of urinary shedders is thus important in preventing spread of leptospirosis. Culturing leptospires from clinical samples is extremely difficult, but molecular techniques, such as qPCR, provide a useful alternative tool for detecting leptospiral shedding. Several studies on renal carriage of leptospires show a wide range of prevalence among asymptomatic dogs. For example, a recent study from Switzerland reported urinary shedding in 0.2% of tested dogs [24], while a group from Brazil reported a prevalence of 19.8% [25]. Other studies have shown prevalence to be 8.2% (USA) [26], 7.05% (Ireland) [27], 14.2% (Brazil) [28], 3.7% (Columbia) [29], 4.8% (Algeria) [30], and 7.6% (New Caledonia) [31].

Classically, *L*. *interrogans* serovars, especially Canicola have been associated with asymptomatic renal carriage in dogs [32]. However, multiple studies have recovered other leptospiral species from asymptomatic dogs, including *L*. *borgpetersenii*, *L*. *kirschneri*, *L*. *wolfii*, and *L*. *santarosai* [33,34,22,35]. In the present study, sequence analysis of PCR-positive dog urine samples revealed that *L*. *interrogans* was the species involved in infections. *L*. *interrogans* has been associated with infection in humans, rodents and other animals [36–39]. Considering the animal and public health significance of these leptospiral species, the role of shelter dogs in transmission cycle in this region should be further investigated.

Leptospiruria or renal carriage of leptospires is not necessarily associated with the seropositivity [40]. The kappa statistics performed on our data showed a fair agreement between qPCR and MAT results, indicating a low correlation. Although it is not surprising, a low correlation between qPCR and MAT results implies that serological tests are not suitable for identification of asymptomatic infected dogs, and these leptospiruric but MAT-negative dogs perhaps pose a greater risk to animal and public health as they are less likely to be detected by routinely recommended serological tests, such as MAT.

A significant number of dogs tested in this study had leptospiral antibodies as detected by MAT. Most of the MAT positive sera were reactive to serovar Icterohaemorrhagiae, followed by serovars Bratislava and Autumnalis. These three serovars have previously been isolated from dogs [39,41]. Association between Icterohaemorrhagiae and rodents is well documented [42]. Since these shelters have limited resources, rodent infestation of the premises is very common in this region. A recent study from our group has shown that 62.3% of rodents in the Cumberland Gap region carry leptospires in their kidneys, potentially contaminating the environment and infecting animals and humans at risk [43].

Earlier studies from different parts of the world have shown urinary shedding and/or seropositivity in cats, suggesting that cats may have a role as a reservoir or accidental host [44–48]. However, in our study, all tested cats were negative for the presence of leptospiral antibodies and leptospiruria. How shelter cats fit in the leptospiral transmission conundrum in this region needs to be reevaluated.

Shelter animals may be exposed to rodents, other wild reservoirs, and stagnant water prior to arriving at shelters or even after entering shelters, if vermin control and disinfection protocols are inadequate. Exposure could also happen when animals come in contact with infected urine of another shelter animal. In a shelter environment, asymptomatic urinary shedders have the potential to infect other animals as well as workers and adopters. Shelter workers are occupationally exposed to dog and cat urine on a daily basis as they provide basic husbandry to these animals. Our study provides evidence-based information to educate shelters on the risk of leptospiral exposure to shelter workers and the importance of vermin control, vaccinating animals, and implementing proper disinfection and hygiene protocols.

Leptospirosis should be on the differential diagnosis list for any animal that has clinical signs such as vomiting, diarrhea, fever, lethargy and anorexia, especially if it has been adopted from a shelter, and prior vaccination status is unknown. Also, prevalence data can help a veterinarian determine an animal’s risk of exposure and the need for vaccination for leptospirosis in their region, since it is a non-core vaccine as per American Animal Health Association Canine Vaccination Guidelines [49]. This study provides veterinarians in this region with supporting evidence to make a case for the need and importance of yearly vaccination of dogs for leptospirosis when discussing preventive care with pet-owners.

## Supporting information

S1 Fig Phylogenetic analysis of the partial nucleotide sequences of the *rpoB* gene of *Leptospira* strains in this study (MarleyKR, MarbleKR, DozerKR, LewisKR, BarneyKR, DarlaKR, HossKR, MirandaKR, BrandiKR, MaryAnnKR, ArielKR, JupiterKR, MandyKR, CindiKR, DaisyKR, GraceKR, HankKR, MeekoKR, CaliKR, HoldenKR and LandonKR) with cognate gene of other leptospiral species (La Scola et al., 2006 [16]). The tree was generated using Geneious 9.0.5.
(PPTX)

S1 Table GenBank accession numbers of leptospiral *rpoB* sequences amplified from positive dog urine. (PPTX)
