# Supplementary material for: Leptospiral shedding and seropositivity in shelter dogs in the Cumberland Gap Region of Southeastern Appalachia
Source: PLoS One. 2020 Jan 30;15(1):e0228038. doi: 10.1371/journal.pone.0228038 (PMC6992200; doi:10.1371/journal.pone.0228038)
Supplement: S1 Fig — The tree was generated using Geneious 9.0.5. (PPTX) [file pone.0228038.s001.pptx]

## Slide 1
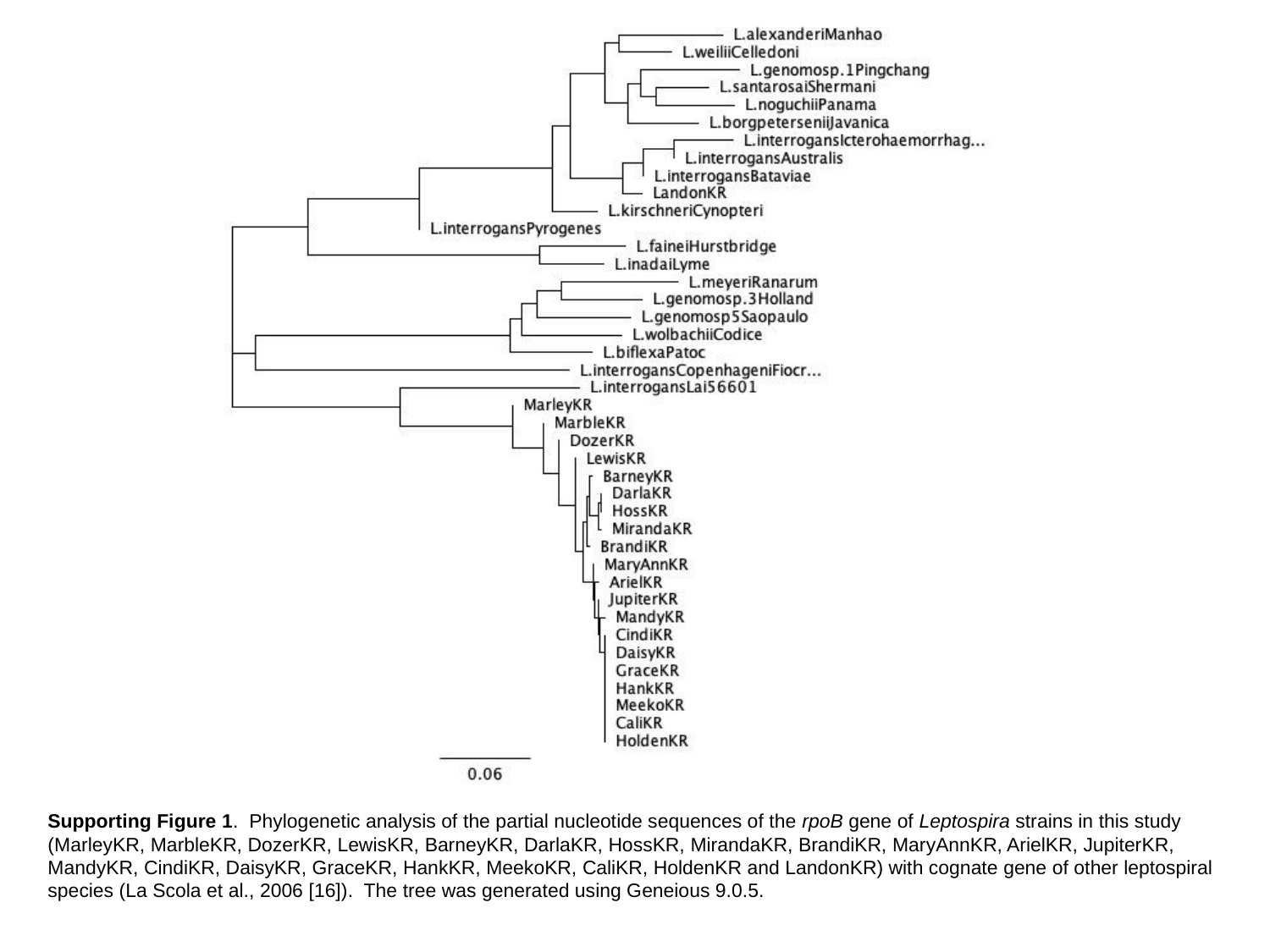

Supporting Figure 1. Phylogenetic analysis of the partial nucleotide sequences of the rpoB gene of Leptospira strains in this study (MarleyKR, MarbleKR, DozerKR, LewisKR, BarneyKR, DarlaKR, HossKR, MirandaKR, BrandiKR, MaryAnnKR, ArielKR, JupiterKR, MandyKR, CindiKR, DaisyKR, GraceKR, HankKR, MeekoKR, CaliKR, HoldenKR and LandonKR) with cognate gene of other leptospiral species (La Scola et al., 2006 [16]). The tree was generated using Geneious 9.0.5.
