# Supplementary material for: Leptospiral shedding and seropositivity in shelter dogs in the Cumberland Gap Region of Southeastern Appalachia
Source: PLoS One. 2020 Jan 30;15(1):e0228038. doi: 10.1371/journal.pone.0228038 (PMC6992200; doi:10.1371/journal.pone.0228038)
Supplement: S1 Table — (PPTX) [file pone.0228038.s002.pptx]

## Slide 1
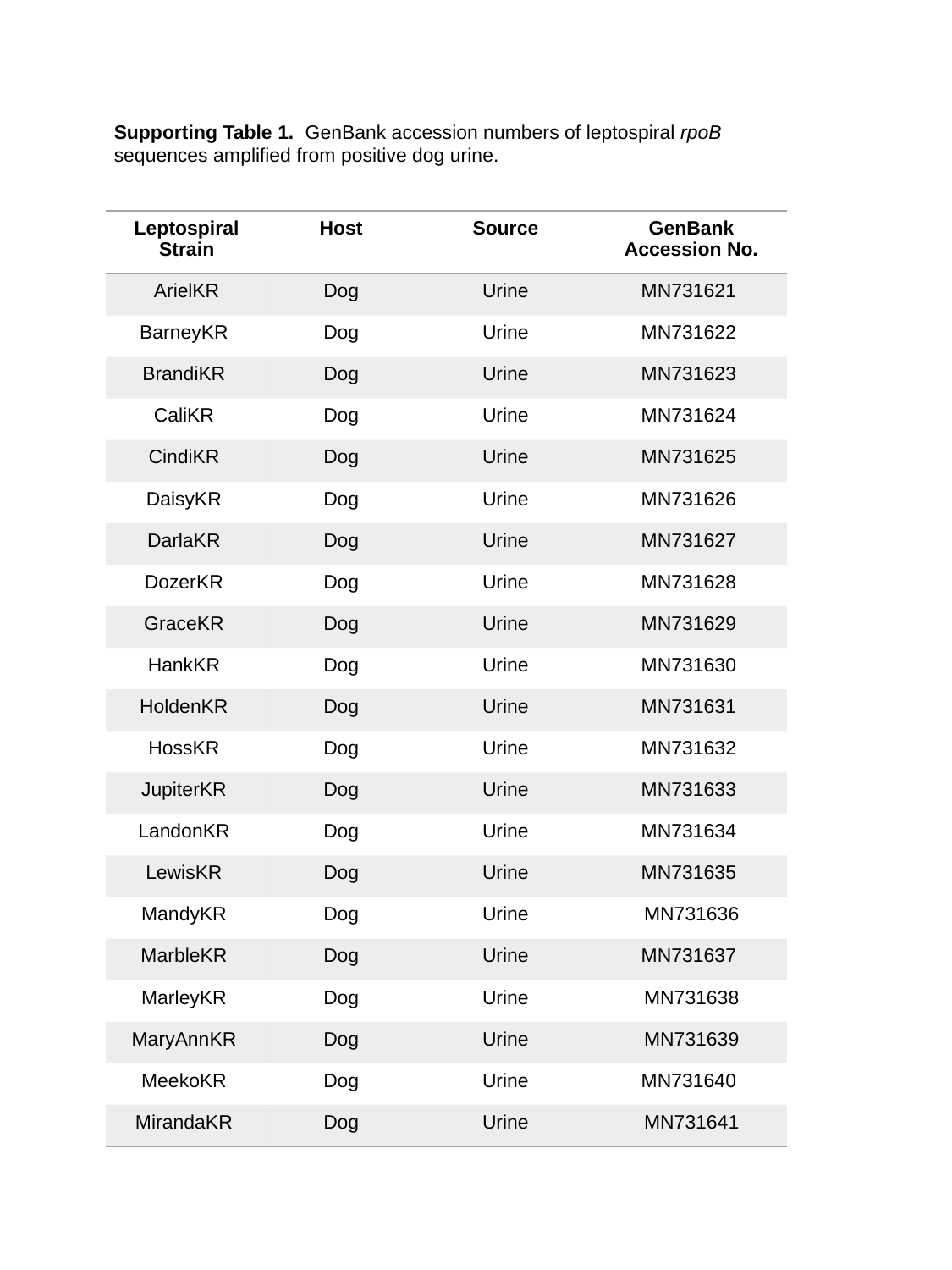

Supporting Table 1. GenBank accession numbers of leptospiral rpoB sequences amplified from positive dog urine.
| Leptospiral Strain | Host | Source | GenBank Accession No. |
| --- | --- | --- | --- |
| ArielKR | Dog | Urine | MN731621 |
| BarneyKR | Dog | Urine | MN731622 |
| BrandiKR | Dog | Urine | MN731623 |
| CaliKR | Dog | Urine | MN731624 |
| CindiKR | Dog | Urine | MN731625 |
| DaisyKR | Dog | Urine | MN731626 |
| DarlaKR | Dog | Urine | MN731627 |
| DozerKR | Dog | Urine | MN731628 |
| GraceKR | Dog | Urine | MN731629 |
| HankKR | Dog | Urine | MN731630 |
| HoldenKR | Dog | Urine | MN731631 |
| HossKR | Dog | Urine | MN731632 |
| JupiterKR | Dog | Urine | MN731633 |
| LandonKR | Dog | Urine | MN731634 |
| LewisKR | Dog | Urine | MN731635 |
| MandyKR | Dog | Urine | MN731636 |
| MarbleKR | Dog | Urine | MN731637 |
| MarleyKR | Dog | Urine | MN731638 |
| MaryAnnKR | Dog | Urine | MN731639 |
| MeekoKR | Dog | Urine | MN731640 |
| MirandaKR | Dog | Urine | MN731641 |
